# Between Autonomy and Paternalism: Attitudes of Nursing Personnel Towards Jehovah’s Witnesses’ Refusal of Blood Transfusion

**DOI:** 10.3389/ijph.2023.1606291

**Published:** 2023-08-03

**Authors:** Jan Domaradzki, Katarzyna Głodowska, Piotr Jabkowski

**Affiliations:** ^1^ Department of Social Sciences and Humanities, Poznań University of Medical Sciences, Poznań, Poland; ^2^ Faculty of Sociology, Adam Mickiewicz University, Poznań, Poland

**Keywords:** acceptance, attitudes, blood transfusion, Jehovah’s Witnesses, nurses’ knowledge, nursing personnel, refusal

## Abstract

**Objectives:** The study describes the attitudes of Polish nursing personnel towards Jehovah’s Witnesses’ (JWs’) refusal to receive blood and blood products.

**Methods:** We developed an online survey assessing nurses’ knowledge and attitudes towards JWs’ refusal of blood transfusion in a life-threatening condition. It also examined nurses’ attitudes towards ethical and legal issues associated with JWs’ refusal of blood transfusions. These questions were explored using a sample of 202 Polish nurses.

**Results:** Nurses’ knowledge of JWs’ stance towards blood transfusions is inadequate and they tended to be ill-disposed towards JWs’ refusal of blood transfusions. Although most nurses respected adult JW patients’ autonomy and supported their right to refuse blood, in the case of JW children they are guided by paternalism. Nurses’ attitudes were affected by whether they had children, whether they declared themselves religious, their level of education and prior experience with patients who had refused a blood transfusion.

**Conclusion:** Since most nurses felt unprepared to care for JW patients, this study reveals an urgent need to train nurses in transcultural nursing and increase nurses’ cultural competencies, and that this should be incorporated into medical *curricula* **.**

## Introduction

Patients’ autonomy, self-determination, choice and consent to treatment are the fundamental ethical principles of patients’ human rights [1]. As such, they are also safeguarded by the *Constitution of the Republic of Poland* [2], which ensures the personal inviolability and security of all Polish citizens (art. 41), and individuals’ right to take decisions regarding their personal life (art. 47). These rights have also been included in the provisions of Polish medical law since 1996 and are regulated by arts 32 and 34 of the *Act on the Professions of Doctors and Dentists* [3], art. 16 of the *Act on Patient`s Rights and the Ombudsman of Patient’s Rights* [4], arts 13 and 15 of the *Code of Medical Ethics* [5] and arts 3b and 3c of *the Code of Professional Ethics of Nurses and Midwives in the Republic of Poland* [*6*]. All these documents emphasise the healthcare professionals’ (HCPs’) duty to obtain patients’ consent to carry out a medical examination or administer nursing treatment. Performing a therapeutic procedure without the patient’s consent is also a criminal offence punishable in art. 192 § 1 of the Polish penal code [7, 8].

At the same time, while by the rules of Polish law and medical ethics HCPs are obliged to treat a patient according to their best will and knowledge, and respect their personal choices (religious), beliefs and values, patients’ personal values and beliefs may cause patients to refuse treatment. One such example occurs among Jehovah’s Witnesses (JWs), well-known to the medical community for their refusal of allogeneic blood transfusion [7–11].

JWs are a Christian denomination founded in the United States in 1872. The church was established in Poland in 1905 and there are currently more than 114,000 adherents in Poland, constituting the third largest denomination in the country [12]. One of the many doctrines adopted by JWs is the prohibition of the consumption of blood or blood products by any route. According to JWs, however, there are medical grounds for this avoidance of blood transfusions. The warrant to abstain from blood is based upon JWs’ interpretation of the Bible (*Genesis* 9:4, *Leviticus* 17:11–14, *Acts* 15:20, 29) [13, 14], which suggests that blood represents life and is sacred to God. Any infringement of the ban is therefore perceived as a violation of God’s law and occasions the risk of thereby foregoing eternal salvation, resulting in potential revoking of one’s membership of the community. JWs therefore often carry a signed and witnessed Advance Decision Document in which they oppose any blood transfusion and list the blood products and autologous procedures they accept.

While JWs are known to refuse allogeneic blood transfusions, many people, including HCPs, are unaware of their regulations pertaining to blood products [15]. Although JWs refuse transfusions of whole blood (including pre-operative autologous donations) and primary blood components, such as red and white cells, platelets and unfractionated plasma, as well as haemoglobin (both natural and recombinant), since 2000 they have been at liberty to take, as acts of “individual conscience,” derivatives of primary blood components, such as haemoglobin-based blood substitutes, interferons, interleukins, albumin, globulins, cryoprecipitate, clotting factor concentrates, including fibrinogen concentrate, and immunoglobulins [9, 15–17].

There tends to be no objection to a number of medical procedures, such as blood donation, transfusions of autologous blood (if kept in continuous circuit with the patient), organ transplantation (if drained of blood), dialysis, intra-operative cell salvage (ICS) and acute normovolemic haemodilution (ANH) which also need to be kept in continuous circuit, apheresis and cardiac bypass. They also allow the use of (non-blood-prime) heart-lung or similar equipment if the extra-corporeal circulation is uninterrupted. Recombinant products such as erythropoiesis-stimulating agents, granulocyte colony-stimulating factors and pharmacological agents (i.e., intravenous iron or tranexamic acid) are also acceptable. While JWs refuse allogeneic blood, they often consent to blood management techniques and procedures that involve a temporary diversion of autologous blood (e.g., autotransfusion), but HCPs still face clinical challenges and ethical and legal dilemmas regarding whether to accede to JW patients’ refusal of a life-saving blood transfusions, a refusal that may result in death [7, 10, 11, 16].

For most HCPs JW patients’ decisions to refuse treatment represents a challenge. Most nurses encounter patients who refuse blood and blood products during their career. Although medical discourse has a great deal by way of ethical and legal considerations on JWs’ refusal to receive blood and blood products [7–9, 18–23], much less is known of the attitudes of nursing personnel (NP) regarding this. This study therefore aims to describe the attitudes of Polish nurses towards JWs’ refusal of blood transfusion.

## Methods

### Study Design

This research included data from an anonymous, self-administered, on-line questionnaire regarding NP’s knowledge and attitudes towards JWs’ refusal of blood transfusions.

### Research Tools

The questionnaire was developed in accordance with the guidelines of the European Statistical System [24]. First, we organised an online focus group of four research experts, comprising a nurse, a sociologist and two Jehovah’s Witnesses, to discuss the questions regarding key issues related to JWs’ refusal of blood transfusion drawn from the literature review. A standardised questionnaire was then developed and pre-tested via an on-line platform with ten nurses, with a re-formulation and re-evaluation of the four questions by three additional external experts in mind: a nurse, a sociologist and a Jehovah’s Witness.

The questionnaire consisted of five sections. The first assessed nurses’ knowledge of JWs’ attitudes towards blood transfusions. The second section included questions regarding nurses’ attitudes to JWs’ refusal of blood transfusions in life-threatening situations. The third section was related to ethical and legal issues associated with JWs’ refusal of blood transfusions. The fourth asked questions regarding nurses’ educational needs on non-blood management techniques. The last section of the questionnaire included questions concerning nurses’ demographic characteristics.

### Participants and Setting

The study was conducted between October 2022 and January 2023 among practicing nurses in Poznan, Poland. While all professionally active nurses were eligible to participate in the survey, respondents were recruited via self-selection from several closed groups for nurses on Facebook.

### Data Collection

After permission to post an invitation to participate in the study was obtained from the moderators of several groups for nurses on Facebook, the final version of the questionnaire was posted on an online platform and distributed among nurses. All participants received an invitation letter and were informed of the study’s purposes, voluntary participation, its anonymous and confidential character, and the right to drop out at any time or to refuse to reveal information regarding their circumstances. Before completing the survey informed consent was obtained from all nurses enrolled. All participants completed self-administered, computer-assisted questionnaires using electronic devices. Completing the questionnaire took approximately 20 min and was collected anonymously.

### Ethical Issues

This study was performed in line with the principles of the Declaration of Helsinki. All HCPs received an invitation letter and informed consent was obtained from all respondents enrolled in the study. Ethics and research governance approval were also obtained from the Poznań University of Medical Sciences (PUMS) Bioethics Committee (KB—760/22).

### Data Analysis

All analyses were performed in the R Project for Statistical Computing [25], and we implemented the following packages for data manipulation, statistical analyses and data visualisation: tidyverse [26], readxl [27], ggplot2 [28], sjPlot [29] and flextable [30].

Whenever we compared the differences in proportions in extracted groups of survey participants, we implemented a two-tail z-test for fractions; (N.B. the z-test statistic tends to be distributed asymptotically with zero-mean and standard deviation equal to 1.) In turn, we performed a chi-squares test to verify the similarity in the distribution of categorical outcomes in extracted groups of respondents.

## Results

Table 1 presents the characteristics of the survey participants: 202 nurses of Polish origin completed the questionnaire. Overall, the gross sample was dominated by women (93.6%), which corresponds to the gender proportion across the nursing population in Poland [31]. The mean age of survey participants was 40 years (range: 22–65), and the mean seniority was 17 years (range: 1–42). Most nurses had a master’s or higher degree (68.8%); 64.4% declared previous experience with a patient who refused a blood transfusion on religious grounds. Most survey participants were married or in a relationship (72.3%), 67.3% had children, 49% declared religious influences in their life-decisions and choices, while 51% declared this to be irrelevant.

**TABLE 1:** Study participants (Poland, 2023).

Characteristics	n	%	Mean	SD
Sex				
Woman	189	93.6		
Man	13	6.4		
Age			40	11.2
Seniority			17	11.9
Education				
Bachelor’s or lower	63	31.2		
Master’s or higher	139	68.8		
Marital status				
Single	35	17.3		
Partnership	24	11.9		
Married	122	60.4		
Widowed	2	1.0		
Divorced	19	9.4		
Do you have children?				
Yes	136	67.3		
No	66	32.7		
Domicile				
Up to 10,000 inhabitants	45	22.3		
10–50,000 inhabitants	47	23.3		
51–100,000 inhabitants	22	10.9		
101–500,000 inhabitants	27	13.4		
Above 500,000 inhabitants	61	30.2		
What role does religion play in your life?				
Significant	47	23.3		
Rather significant	52	25.7		
Little	66	32.7		
None	37	18.3		
During your professional career, have you ever been in a situation in which a person has refused an allogeneic blood transfusion due to his or her religious beliefs?				
Yes	130	64.4		
No	72	35.6		

**FIGURE 1:**
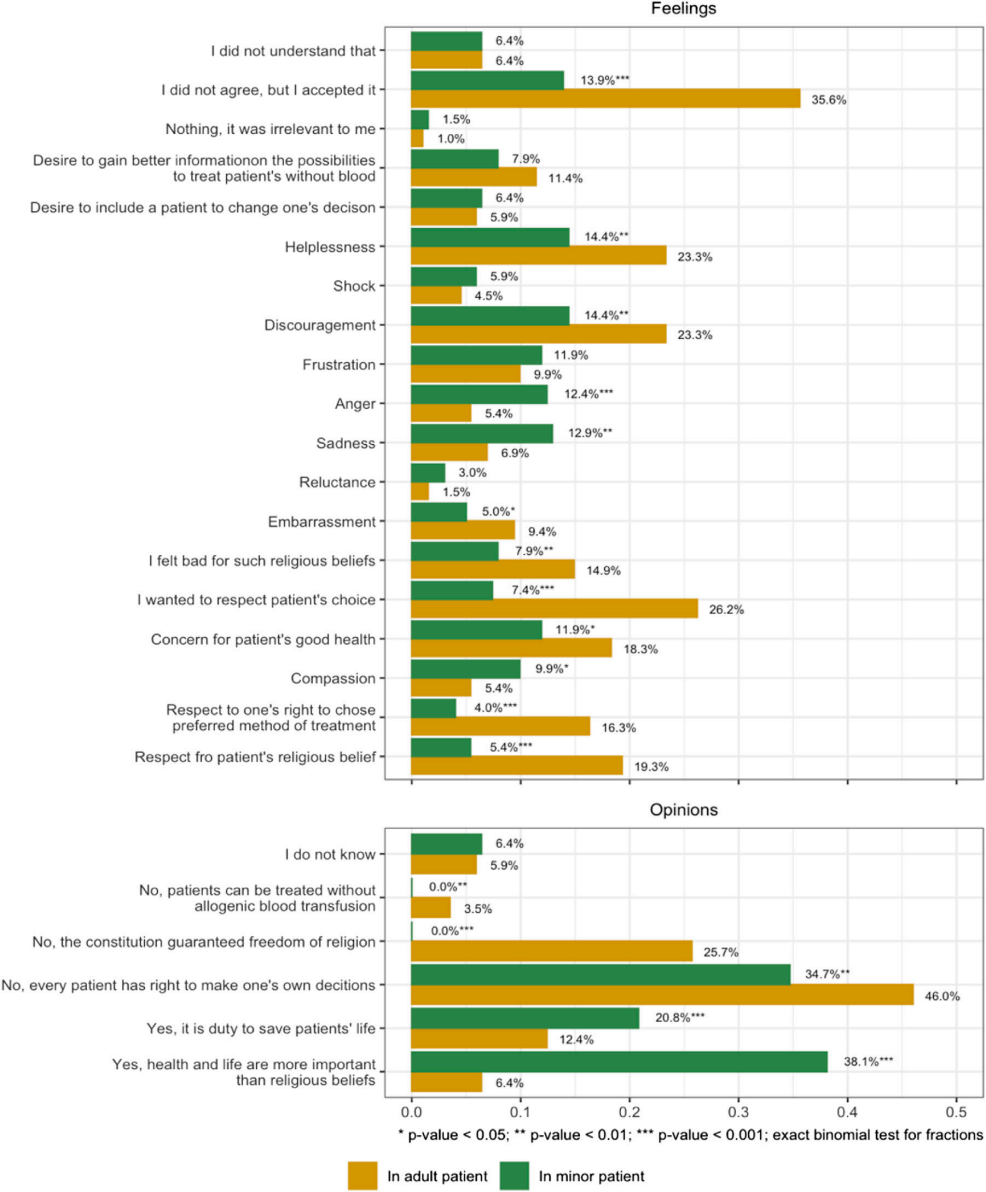
Nurses’ feelings and opinions on Jehovah’s Witness patients’ refusal of blood transfusion is life-threatening circumstances (Poland, 2023).

The upper panel of Figure 1 presents NP’s feelings regarding JWs’ refusal of blood transfusion as a threat to life; the *x*-axis demonstrates the proportion of NP’s that agree with the statements included on the *y*-axis. It shows significant differences in respondents’ emotional reactions towards adult patients and the children of JWs. Regarding adult JW patients NP’s feelings were dominated by acceptance and lack of understanding (35.6% vs. 13.9%), respect for patients’ choices (26.2% vs. 7.4%), including their right to choose a preferred method of treatment (16.3% vs. 4%) and respect for their religious beliefs (19.3% vs. 5.4%), but also helplessness and discouragement (23.3% vs. 14.4%). In contrast, in the case of juvenile JWs three feelings predominated: sadness (12.9% vs. 6.9%), anger (12.4% vs. 5.4%) and feeling that such religious beliefs were wrong (14.9% vs. 7.9%)**.**

The lower panel of Figure 1 demonstrates differences in NP’s opinions on whether, in a life-threatening situation, HCPs should perform a blood transfusion on JW patients, adult and juvenile alike, with neither the patients’ knowledge nor consent. In the case of an adult JW most nurses rejected the idea of performing a blood transfusion without the patient’s knowledge, referring to their freedom of conscience and religion guaranteed by the Polish constitution and stressing the patient’s right to make autonomous decisions and choices to be treated without allogeneic blood transfusion (in total 75.2%). However, 58.9% of NP believed that in the case of JW children blood transfusion should be given without patients’ permission, since it is HCPs duty to save a patient’s life or because they believed that saving patients’ lives takes precedence over their religious beliefs.

**FIGURE 2:**
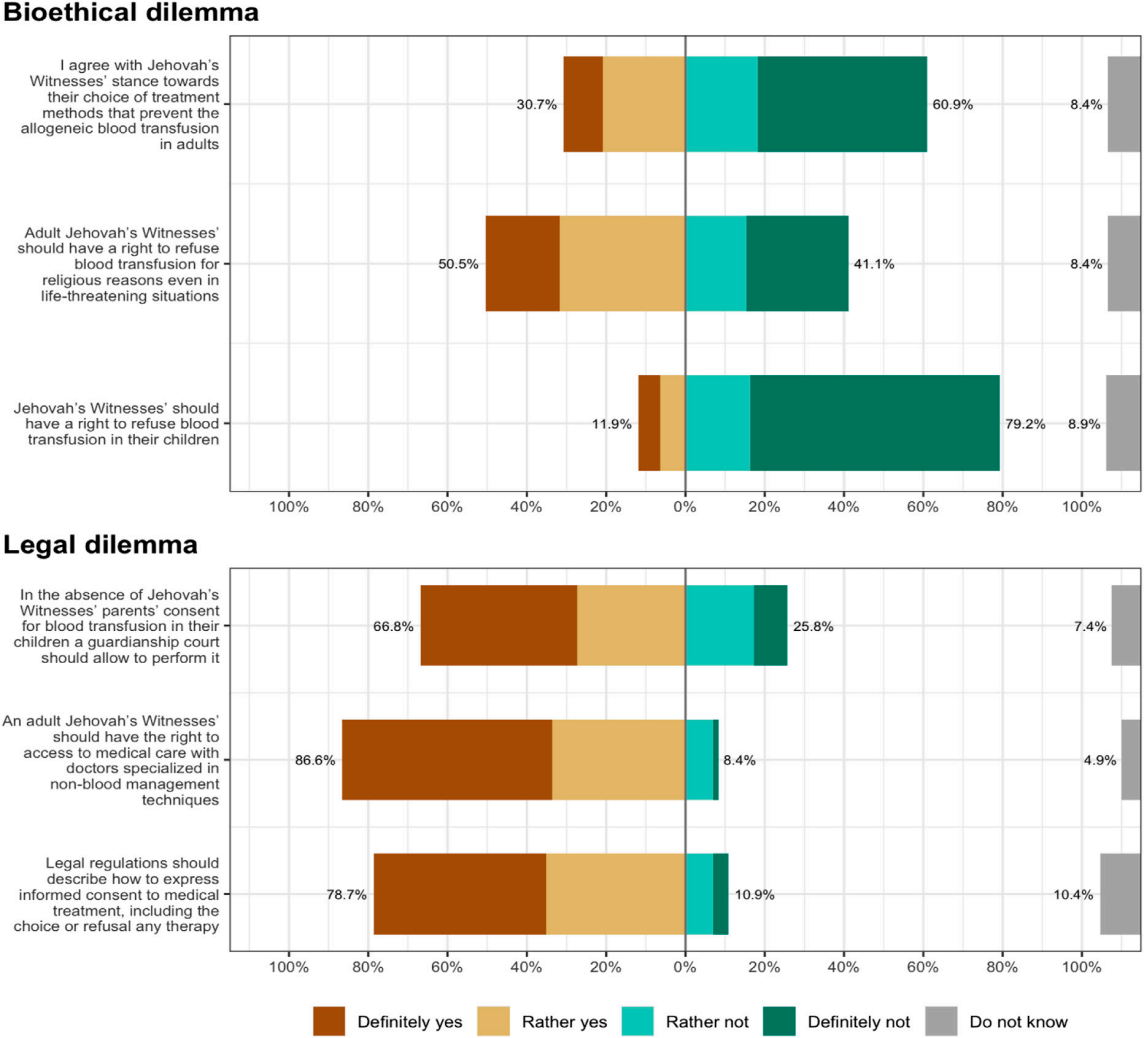
Bioethical and legal dilemmas related to Jehovah’s Witnesses’ stance towards blood transfusion (Poland, 2023).

Figure 2 also presents NP’s opinions on the bioethical and legal dilemmas of blood transfusion in a JW patient. While most nurses rejected JW children’s right to refuse blood transfusions (79.2%) and showed no understanding of JWs’ stance toward blood transfusion methods (60.9%), half of the participants supported adult JWs’ rights to refuse blood transfusion on religious grounds (50.5%). On the other hand, while most nurses believed that an adult JW patient should have access to medical care with non-blood management techniques (86.6%), they also declared that, should parents’ consent be lacking, the guardianship court should allow blood transfusion in JW children (66.8%). At the same time, most participants believed that the decision to refuse treatment should be regulated by law (78.7%).

The differences in NP’s perception of bioethical and legal dilemmas (i.e., the fraction of NP’s that agree with the following statements) in groups based on socio-demographic characteristics are presented in Table 2. Despite no apparent pattern in the impact of selected socio-demographics, some interesting differences were observed. Firstly, nurses who had children (z-value = 2.81; *p* = 0.005) and declared themselves to be religious (z-value = 5.43; *p* < 0.001) supported JWs’ right to refuse blood transfusions on behalf of their children more often. Secondly, NP with a higher education degree (z-value = 3.81; *p* < 0.001) and those less religious (z-value = 2.25; *p* = 0.025) were more willing to support JWs’ right to refuse a blood transfusion on religious grounds. Thirdly, nurses who had previous experience of patients’ refusing blood transfusions were more eager to support JWs’ right to medical care with non-blood techniques (z-value = 2.14; *p* = 0.032). They also wanted precise legal regulations on expressing informed consent for medical treatment more often (z-value = 3.50; *p* < 0.001).

**TABLE 2:** Bioethical and legal dilemmas related to Jehovah’s Witnesses’ stance on blood transfusion according to socio-demographic characteristics (Poland, 2023).

Statement	Overall proportion of nurses who agree with the statement	Bachelor’s or lower	Master’s or higher	z-value	Proportion with children	Proportion without children	z-value	Not religious	Religious	z-value	Prior experience of refusal	No experience of refusal	z-value
Bioethical dilemma													
1) I agree with Jehovah’s Witnesses’ stance regarding the refusal of treatment methods that involve allogeneic blood transfusion in adults	0.307	0.270	0.324	1.66 (*p* = 0.097)	0.309	0.303	0.18 (*p* = 0.858)	0.282	0.333	1.60 (*p* = 0.111)	0.315	0.292	0.73 (*p* = 0.465)
2) Adult Jehovah’s Witnesses should have the right to refuse blood transfusions for religious reasons even in life-threatening circumstances	0.505	0.413	0.547	**3.81 (*p* < 0.001)**	0.515	0.485	0.85 (*p* = 0.396)	0.544	0.465	**2.25 (*p* = 0.025)**	0.515	0.486	0.83 (*p* = 0.405)
3) Jehovah’s Witnesses should have the right to refuse blood transfusions on behalf of their children	0.119	0.095	0.129	1.50 (*p* = 0.132)	0.140	0.076	**2.81 (*p* = 0.005)**	0.058	0.182	**5.43 (*p* < 0.001)**	0.131	0.097	1.47 (*p* = 0.141)
Legal dilemma													
1) In the absence of parental consent for blood transfusions for Jehovah’s Witness children a guardianship court should be able to grant a doctor permission to perform it	0.668	0.698	0.655	1.32 (*p* = 0.187)	0.669	0.667	0.07 (*p* = 0.941)	0.757	0.576	**5.48 (*p* < 0.001)**	0.654	0.694	1.23 (*p* = 0.22)
2) An adult Jehovah’s Witnesses should have the right to medical care from doctors specialised in non-blood management techniques	0.866	0.889	0.856	1.37 (*p* = 0.171)	0.882	0.833	**2.05 (*p* = 0.041)**	0.874	0.859	0.63 (*p* = 0.526)	0.885	0.833	**2.14 (*p* = 0.032)**
3) Legal regulations should describe the way to express informed consent for medical treatment, including the choice or refusal of any particular therapy	0.787	0.778	0.791	0.47 (*p* = 0.637)	0.772	0.818	1.60 (*p* = 0.109)	0.786	0.788	0.05 (*p* = 0.959)	0.823	0.722	**3.50 (*p* < 0.001)**

Notes: Statistically significant differences are written in boldface.

Table 3 presents the proportion of correct answers to the questions seeking to ascertain NPs’ knowledge of JWs’ stance on blood transfusions. For the majority of items, the proportion of correct answers was between 60%–80%, indicating a relatively high level of knowledge. The proportion of correct answers in several items was markedly below 50%, indicating a lack of knowledge in specific areas. Socio-demographic characteristics also influenced NP’s levels of knowledge: NP with a higher educational degree, who declared themselves more religious, had children and prior experience of a patient who had refused a blood transfusion tended more frequently to give correct answers.

**TABLE 3:** Nurses’ knowledge by socio-demographic characteristics (Poland, 2023).

Statement with correct answer in a bracket	Correct answers	Bachelor’s or lower	Master’s or higher	z-value	Proportion with children	Proportion without children	z-value	Not religious	Religious	z-test	Prior experience of refusal	No experience of experienced	z-value
Jehovah’s Witnesses’ refusal of allogeneic blood transfusion concerns													
Red blood cells (yes)	0.317	0.365	0.295	**2.14 (*p* = 0.032)**	0.331	0.288	1.31 (*p* = 0.189)	0.340	0.293	1.43 (*p* = 0.152)	0.369	0.222	**4.49 (*p* < 0.001)**
White blood cells (yes)	0.243	0.254	0.237	0.55 (*p* = 0.583)	0.272	0.182	**2.99 (*p* = 0.003)**	0.272	0.212	**1.98 (*p* = 0.048)**	0.292	0.153	**4.63 (*p* < 0.001)**
Platelets (yes)	0.248	0.286	0.230	1.83 (*p* = 0.068)	0.272	0.197	**2.47 (*p* = 0.013)**	0.291	0.202	**2.94 (*p* = 0.003)**	0.292	0.167	**4.14 (*p* < 0.001)**
Fractions from red blood cells (no)	0.777	0.762	0.784	0.76 (*p* = 0.447)	0.765	0.803	1.31 (*p* = 0.191)	0.738	0.818	**2.74 (*p* = 0.006)**	0.723	0.875	**5.19 (*p* < 0.001)**
Fractions from white blood cells (no)	0.832	0.825	0.835	0.35 (*p* = 0.729)	0.824	0.848	0.95 (*p* = 0.343)	0.777	0.889	**4.26 (*p* < 0.001)**	0.792	0.903	**4.20 (*p* < 0.001)**
Platelet-derived fractions (no)	0.752	0.714	0.770	1.83 (*p* = 0.068)	0.728	0.803	**2.47 (*p* = 0.013)**	0.709	0.798	**2.94 (*p* = 0.003)**	0.708	0.833	**4.14 (*p* < 0.001)**
Plasma-derived fractions (no)	0.881	0.873	0.885	0.52 (*p* = 0.602)	0.875	0.894	0.83 (*p* = 0.405)	0.864	0.899	1.53 (*p* = 0.125)	0.854	0.931	**3.37 (*p* < 0.001)**
Jehovah’s Witnesses’ refusal of blood transfusion concerns													
Pre-operative autologous blood donation for re-infusion (yes)	0.307	0.238	0.338	**3.08 (*p* = 0.002)**	0.331	0.258	**2.26 (*p* = 0.024)**	0.311	0.303	0.24 (*p* = 0.814)	0.323	0.278	1.40 (*p* = 0.163)
Taking white blood cells (yes)	0.213	0.222	0.209	0.47 (*p* = 0.637)	0.235	0.167	**2.38 (*p* = 0.017)**	0.243	0.182	**2.11 (*p* = 0.034)**	0.231	0.181	1.74 (*p* = 0.081)
Acute normovolemic haemodilution (no)	0.886	0.921	0.871	**2.24 (*p* = 0.025)**	0.875	0.909	1.53 (*p* = 0.127)	0.845	0.929	**3.79 (*p* < 0.001)**	0.869	0.917	**2.12 (*p* = 0.034)**
Dialysis (no)	0.926	0.905	0.935	1.65 (*p* = 0.098)	0.949	0.879	**3.78 (*p* < 0.001)**	0.883	0.970	**4.67 (*p* < 0.001)**	0.923	0.931	0.41 (*p* = 0.685)
Extracorporeal circulation (no)	0.886	0.889	0.885	0.18 (*p* = 0.858)	0.904	0.848	**2.50 (*p* = 0.012)**	0.835	0.939	**4.67 (*p* < 0.001)**	0.885	0.889	0.19 (*p* = 0.848)
Intra-operative blood recovery (no)	0.896	0.857	0.914	**2.63 (*p* = 0.008)**	0.890	0.909	0.90 (*p* = 0.367)	0.874	0.919	**2.11 (*p* = 0.034)**	0.877	0.931	**2.50 (*p* = 0.013)**
Jehovah’s Witnesses accept													
Laboratory tests of autologous blood (yes)	0.931	0.937	0.928	0.47 (*p* = 0.636)	0.934	0.924	0.54 (*p* = 0.592)	0.922	0.939	0.95 (*p* = 0.34)	0.954	0.889	**3.64 (*p* < 0.001)**
Laboratory tests of allogeneic blood (yes)	0.916	0.905	0.921	0.82 (*p* = 0.41)	0.912	0.924	0.64 (*p* = 0.523)	0.932	0.899	1.69 (*p* = 0.091)	0.946	0.861	**4.35 (*p* < 0.001)**
Administration of local anaesthesia (yes)	0.946	0.937	0.950	0.82 (*p* = 0.411)	0.963	0.909	**3.39 (*p* < 0.001)**	0.942	0.949	0.49 (*p* = 0.627)	0.977	0.889	**5.51 (*p* < 0.001)**
Administration of general anaesthesia (yes)	0.931	0.921	0.935	0.82 (*p* = 0.413)	0.963	0.864	**5.57 (*p* < 0.001)**	0.922	0.939	0.95 (*p* = 0.34)	0.954	0.889	**3.64 (*p* < 0.001)**
Protective vaccinations (yes)	0.718	0.714	0.719	0.16 (*p* = 0.871)	0.713	0.727	0.44 (*p* = 0.658)	0.709	0.727	0.59 (*p* = 0.558)	0.708	0.736	0.90 (*p* = 0.369)
Bone marrow transplant (yes)	0.129	0.048	0.165	**5.00 (*p* < 0.001)**	0.125	0.136	0.48 (*p* = 0.63)	0.126	0.131	0.22 (*p* = 0.829)	0.108	0.167	**2.50 (*p* = 0.012)**
Orthopaedic procedures (yes)	0.861	0.857	0.863	0.25 (*p* = 0.8)	0.882	0.818	**2.64 (*p* = 0.008)**	0.835	0.889	**2.22 (*p* = 0.027)**	0.885	0.819	**2.68 (*p* = 0.007)**
Surgical procedures involving autologous blood (yes)	0.371	0.254	0.424	**5.02 (*p* < 0.001)**	0.316	0.485	**4.96 (*p* < 0.001)**	0.417	0.323	**2.77 (*p* = 0.006)**	0.377	0.361	0.47 (*p* = 0.642)
Organ transplantation involving autologous blood (yes)	0.173	0.111	0.201	**3.39 (*p* < 0.001)**	0.132	0.258	**4.70 (*p* < 0.001)**	0.175	0.172	0.11 (*p* = 0.909)	0.185	0.153	1.20 (*p* = 0.232)
Pre-operative autologous blood donation for re-infusion (no)	0.446	0.460	0.439	0.61 (*p* = 0.539)	0.471	0.394	**2.19 (*p* = 0.028)**	0.398	0.495	**2.77 (*p* = 0.006)**	0.469	0.403	1.90 (*p* = 0.057)
Organ donation (yes)	0.149	0.127	0.158	1.25 (*p* = 0.211)	0.169	0.106	**2.52 (*p* = 0.012)**	0.165	0.131	1.35 (*p* = 0.178)	0.177	0.097	**3.19 (*p* = 0.001)**
Plasmapheresis (yes)	0.188	0.095	0.230	**4.91 (*p* < 0.001)**	0.176	0.212	1.30 (*p* = 0.195)	0.184	0.192	0.27 (*p* = 0.786)	0.185	0.194	0.36 (*p* = 0.721)
Cell labelling (yes)	0.104	0.095	0.108	0.59 (*p* = 0.555)	0.103	0.106	0.15 (*p* = 0.885)	0.107	0.101	0.27 (*p* = 0.788)	0.085	0.139	**2.53 (*p* = 0.011)**
Using epidural blood patch (yes)	0.238	0.222	0.245	0.75 (*p* = 0.455)	0.206	0.303	**3.24 (*p* = 0.001)**	0.262	0.212	1.67 (*p* = 0.095)	0.246	0.222	0.80
Using of autologous platelet-rich gel (yes)	0.228	0.254	0.216	1.29 (*p* = 0.196)	0.213	0.258	1.50 (*p* = 0.133)	0.204	0.253	1.65 (*p* = 0.099)	0.192	0.292	3.37***
Stem cell transplant (yes)	0.139	0.079	0.165	**3.54 (*p* < 0.001)**	0.132	0.152	0.79 (*p* = 0.431)	0.117	0.162	1.86 (*p* = 0.064)	0.138	0.139	0.02
Honorary blood donation (no)	0.713	0.651	0.741	**2.83 (*p* = 0.005)**	0.699	0.742	1.38 (*p* = 0.168)	0.728	0.697	0.98 (*p* = 0.327)	0.754	0.639	3.61***
Jehovah’s Witnesses’ prohibition of blood transfusion applies to													
Infants before the first week of life (yes)	0.748	0.778	0.734	1.44 (*p* = 0.15)	0.787	0.667	**3.93 (*p* < 0.001)**	0.777	0.717	1.95 (*p* = 0.051)	0.762	0.722	1.29 (*p* = 0.198)
Children (yes)	0.807	0.825	0.799	0.97 (*p* = 0.334)	0.824	0.773	1.83 (*p* = 0.067)	0.806	0.808	0.08 (*p* = 0.935)	0.846	0.736	**3.96 (*p* < 0.001)**
Pregnant women (yes)	0.817	0.825	0.813	0.46 (*p* = 0.647)	0.838	0.773	**2.41 (*p* = 0.016)**	0.825	0.808	0.63 (*p* = 0.528)	0.854	0.750	**3.82 (*p* < 0.001)**
People above 75 years of age (yes)	0.827	0.825	0.827	0.07 (*p* = 0.942)	0.853	0.773	**3.01 (*p* = 0.003)**	0.825	0.828	0.11 (*p* = 0.909)	0.854	0.778	**2.86 (*p* = 0.004)**
People with disabilities (yes)	0.812	0.825	0.806	0.71 (*p* = 0.475)	0.838	0.758	**2.93 (*p* = 0.003)**	0.825	0.798	0.99 (*p* = 0.321)	0.838	0.764	**2.71 (*p* = 0.007)**
People on dialysis (yes)	0.723	0.730	0.719	0.34 (*p* = 0.733)	0.721	0.727	0.21 (*p* = 0.832)	0.728	0.717	0.35 (*p* = 0.727)	0.731	0.708	0.71 (*p* = 0.476)
People with blood disease, i.e., haemophilia (yes)	0.743	0.762	0.734	0.91 (*p* = 0.361)	0.779	0.667	**3.67 (*p* < 0.001)**	0.767	0.717	1.62 (*p* = 0.105)	0.762	0.708	1.73 (*p* = 0.084)
People who have to take immunoglobulins (yes)	0.668	0.698	0.655	1.32 (*p* = 0.187)	0.684	0.636	1.43 (*p* = 0.152)	0.699	0.636	1.89 (*p* = 0.059)	0.685	0.639	1.38 (*p* = 0.167)
All Jehovah’s Witnesses without exception (yes)	0.837	0.841	0.835	0.26 (*p* = 0.796)	0.890	0.727	**6.24 (*p* < 0.001)**	0.854	0.818	1.39 (*p* = 0.164)	0.862	0.792	**2.69 (*p* = 0.007)**

Notes: Statistically significant differences are written in boldface.

Finally, Table 4 presents NP’s educational needs on non-blood management techniques. While most nurses rated their knowledge as insufficient or poor (87.2%), two-thirds felt unprepared to care for a patient requiring non-blood management techniques (66.3%). 65.8% of nurses had undergone no instruction in non-blood management techniques and the provision of care to patients who refuse a blood transfusion, and 88.1% expressed the desire to broaden their knowledge in this respect. Additionally, 86.2% believed *medical curricula* should include a mandatory course on strategies to minimise blood loss during surgical procedures in order to minimise the need for blood transfusion. No statistically significant differences in educational needs by socio-demographic characteristics were found, suggesting an urgent need to improve the knowledge and cultural competence of all Polish nurses.

**TABLE 4:** Nurses’ educational needs on non-blood management techniques by socio-demographic characteristics: number of indications and percentages in brackets (Poland, 2023).

Question	Total	Bachelor’s or lower	Master’s or higher	Proportion with children	Proportion without children	Ambivalent/non-religious	Religious	Prior experience of refusal	No experience of refusal
How would you rate your knowledge of non-blood management techniques?									
Fair enough^a^	26 (12.9%)	11 (17.5%)	15 (10.8%)	19 (14.0%)	7 (10.6%)	9 (8.7%)	17 (17.2%)	18 (13.8%)	8 (11.1%)
Insufficient	109 (54.0%)	30 (47.6%)	79 (56.8%)	77 (56.6%)	32 (48.5%)	56 (54.4%)	53 (53.5%)	66 (50.8%)	43 (59.7%)
Very poor	67 (33.2%)	22 (34.9%)	45 (32.4%)	40 (29.4%)	27 (40.9%)	38 (36.9%)	29 (29.3%)	46 (35.4%)	21 (29.2%)
Chi-square test	—	$X^{2}$ = 2.26 (df = 2) (*p* = 0.320)		$X^{2}$ = 2.71 (df = 2) (*p* = 0.260)		$X^{2}$ = 3.68 (df = 2) (*p* = 0.160)		$X^{2}$ = 1.50 (df = 2) (*p* = 0.470)	
Did you have any classes on non-blood management techniques (bloodless medicine) that involve strategies for avoiding blood transfusion and providing care to patients who refuse a blood transfusion?									
Yes	29 (14.4%)	7 (11.1%)	22 (15.8%)	19 (14.0%)	10 (15.2%)	13 (12.6%)	16 (16.2%)	13 (10.0%)	16 (22.2%)
No	133 (65.8%)	46 (73.0%)	87 (62.6%)	90 (66.2%)	43 (65.2%)	75 (72.8%)	58 (58.6%)	92 (70.8%)	41 (56.9%)
I do not know	40 (19.8%)	10 (15.9%)	30 (21.6%)	27 (19.9%)	13 (19.7%)	15 (14.6%)	25 (25.3%)	25 (19.2%)	15 (20.8%)
Chi-square test	—	$X^{2}$ = 2.10 (df = 2) (*p* = 0.350)		$X^{2}$ = 0.05 (df = 2) (*p* = 0.970)		$X^{2}$ = 4.91 (df = 2) (*p* = 0.090)		$X^{2}$ **= 6.23 (df = 2) (*p* = 0.040)**	
Would you like to extend your knowledge regarding non-blood management techniques?									
definitely yes	79 (39.1%)	22 (34.9%)	57 (41.0%)	55 (40.4%)	24 (36.4%)	38 (36.9%)	41 (41.4%)	52 (40.0%)	27 (37.5%)
Rather yes	99 (49.0%)	30 (47.6%)	69 (49.6%)	67 (49.3%)	32 (48.5%)	54 (52.4%)	45 (45.5%)	63 (48.5%)	36 (50.0%)
Rather no^b^	18 (8.9%)	7 (11.1%)	11 (7.9%)	10 (7.4%)	8 (12.1%)	9 (8.7%)	9 (9.1%)	11 (8.5%)	7 (9.7%)
I do not know	6 (3.0%)	4 (6.3%)	2 (1.4%)	4 (2.9%)	2 (3.0%)	2 (1.9%)	4 (4.0%)	4 (3.1%)	2 (2.8%)
Chi-square test	—	$X^{2}$ = 4.46 (df = 3) (*p* = 0.220)		$X^{2}$ = 1.33 (df = 3) (*p* = 0.720)		$X^{2}$ = 1.52 (df = 3) (*p* = 0.680)		$X^{2}$ = 0.19 (df = 3) (*p* = 0.980)	
Do you think there should be a mandatory course on strategies to minimise blood loss during surgery and prevent blood transfusion (patient blood management and non-blood management techniques) in medical curricula?									
Definitely yes	85 (42.1%)	26 (41.3%)	59 (42.4%)	57 (41.9%)	28 (42.4%)	42 (40.8%)	43 (43.4%)	54 (41.5%)	31 (43.1%)
Rather yes	89 (44.1%)	29 (46.0%)	60 (43.2%)	63 (46.3%)	26 (39.4%)	49 (47.6%)	40 (40.4%)	55 (42.3%)	34 (47.2%)
Rather no	17 (8.4%)	4 (6.3%)	13 (9.4%)	10 (7.4%)	7 (10.6%)	8 (7.8%)	9 (9.1%)	12 (9.2%)	5 (6.9%)
Definitely no	1 (0.5%)	0 (0.0%)	1 (0.7%)	0 (0.0%)	1 (1.5%)	1 (1.0%)	0 (0.0%)	0 (0.0%)	1 (1.4%)
I do not know	10 (5.0%)	4 (6.3%)	6 (4.3%)	6 (4.4%)	4 (6.1%)	3 (2.9%)	7 (7.1%)	9 (6.9%)	1 (1.4%)
Chi-square test	—	$X^{2}$ = 1.37 (df = 4) (*p* = 0.850)		$X^{2}$ = 3.35 (df = 4) (*p* = 0.500)		$X^{2}$ = 3.50 (df = 4) (*p* = 0.480)		$X^{2}$ = 5.24 (df = 4) (*p* = 0.260)	
Do you feel prepared to care for a patient who requires treatment with non-blood management techniques?									
Definitely yes	7 (3.5%)	3 (4.8%)	4 (2.9%)	6 (4.4%)	1 (1.5%)	3 (2.9%)	4 (4.0%)	5 (3.8%)	2 (2.8%)
Rather yes	51 (25.2%)	17 (27.0%)	34 (24.5%)	41 (30.1%)	10 (15.2%)	18 (17.5%)	33 (33.3%)	35 (26.9%)	16 (22.2%)
Rather no	91 (45.0%)	22 (34.9%)	69 (49.6%)	55 (40.4%)	36 (54.5%)	51 (49.5%)	40 (40.4%)	57 (43.8%)	34 (47.2%)
Definitely no	43 (21.3%)	16 (25.4%)	27 (19.4%)	28 (20.6%)	15 (22.7%)	27 (26.2%)	16 (16.2%)	28 (21.5%)	15 (20.8%)
I do not know	10 (5.0%)	5 (7.9%)	5 (3.6%)	6 (4.4%)	4 (6.1%)	4 (3.9%)	6 (6.1%)	5 (3.8%)	5 (6.9%)
Chi-square test	—	$X^{2}$ = 5.01 (df = 4) (*p* = 0.290)		$X^{2}$ = 7.34 (df = 4) (*p* = 0.120)		$X^{2}$ = 9.02 (df = 4) (*p* = 0.060)		$X^{2}$ = 1.58 (df = 4) (*p* = 0.810)	

^a^ Notes: Nobody indicated the answer “Very good.”

^b^ Nobody indicated the answer “Definitely no”; Statistically significant differences are written in boldface.

## Discussion

All medical intervention, including blood transfusions, requires patients’ informed voluntary consent. Since the introduction of the blood ban in 1945, the treatment of JWs has raised ethical and legal dilemmas for all HCPs, who feel torn between their duty to respect patients’ autonomy and safeguard their health and their duty to save life at all costs. Some, however, argue that from a medical point of view, JWs’ stance on blood transfusion is irrational, so HCPs should reject JWs’ decision without making a moral judgment. It is also suggested that HCPs’ approach to JW patients should be based on the so-called rational non-interventional paternalism, i.e., they should form their opinions of what is best for JW patients and argue rationally with them [18, 19, 32]. Adopting a *don’t-ask-don’t-tell* policy regarding JWs’ medical care, which is against the religious group’s doctrine and assumes that JWs should be neither asked about nor insist they disclose their personal medical information, either to one another or to the church organisation, is also recommended [20, 21]. Others reject these arguments and claim that the clash of JWs’ values and those held by HCPs do not implicitly make their decision wrong and that the assessment of JWs’ competence should be based on their ability to make decisions rather than on the decisions themselves [33, 34]. At the same time, research shows that, as Polish physicians and nurses face ethical and other non-medical difficulties in making clinical decisions, many are still embedded in a paternalistic tradition of practicing medicine [35, 36].

Of equal importance is that JWs’ refusal of potentially life-saving blood transfusion treatment is a constitutionally protected right recognised in many jurisdictions. The Polish courts have also ruled out that competent adult JW patients’ have the right to decline a doctor’s recommendation and they must be allowed to refuse blood transfusion. One such decision involved a patient (female, initials B.Ł.) who lost consciousness in a traffic accident on 18th August 2004 and required a blood transfusion on account of her injuries. As she was a JW and carried her “Healthcare Statement—No Blood,” in which she rejected all forms of blood transfusion, the doctor asked the District Court to authorise the performance of a blood transfusion to save her life, which he was acceded to. Once she recovered, B.Ł. sued the hospital for acting against her will and in 2005 the Supreme Court ruled that the patient’s *pro futuro* statement was legally binding regarding medical professionals in the case of loss of consciousness and allowed B.Ł.’s complaint [37]. A similar situation occurred in 2020, when the Provincial Administrative Court ruled that the hospital that performed a blood transfusion against a patient’s will in 2017 violated his right to refuse treatment that may not be forced upon anybody [38].

On the other hand, although Western courts recognise parental rights, it is often argued that these rights are not absolute and a child’s health, safety and welfare should always come first. Consequently, when parental refusal of a blood transfusion may put a JW child’s life in danger, a court may be asked to intervene and allow the transfusion in order to protect the child’s welfare [39]. In Poland, if a JW parent absolutely objects to a blood transfusion that might safeguard a child’s health or save its life, a legal procedure that allows the medical personnel to administer blood may be initiated [40]. It rests on art. 111 of the *Family and guardianship code*, which declares that parents may be divested of parental responsibility by the court [41].

It should also be noted that Poland is a religiously uniform society, as an overwhelming majority of Poles are Catholic [42]. Most Poles therefore know no members of other denominations. At the same time, although JWs are the most well-known religious minority in Poland, 60% of Poles claiming to know a JW personally, most Poles still display a greater social distance from JWs than other Christian denominations, including adherents of Eastern Orthodoxy, Protestantism and Judaism [43]. Similar results were found in a recent study on the attitudes of Polish nurses towards followers of various religions, which showed moderate social distance from JWs. It also confirmed our findings that NPs’ age, seniority, prior contacts with other religions and declared religiousness clearly affect social distance from JWs [44]**.**

This research therefore shows that, while most NPs supported adult JW patients’ right to refuse a blood transfusion, they showed little understanding for such a decision and expressed resentment towards JWs’ stance. A study by Jakubowska et al. [45] also showed that 50.02% of paediatric nurses in Lublin (eastern Poland) disagreed with JWs concerning blood treatment. Gouezec et al. [46], on the other hand, demonstrated that, although French doctors do not oppose the medical care of JWs, the majority were somewhat lacking in their awareness of all the regulatory requirements, and remained committed to their primary focus: to save the patient, as long as it is not an end-of-life situation. Finally, a study by Rajtar [47] demonstrated that, while JWs in Germany claimed autonomy based on choice, German physicians often claimed autonomy based on reason.

We also found a significant difference between NP’s attitudes towards adult and juvenile JW patients. Since most NPs in this study believed that it is HCPs’ duty to save a patient’s life, most claimed that a blood transfusion on a juvenile JW patient should be administered even without the patient’s consent. This finding is in line with the observation made by others in that HCPs are more likely to give a transfusion to an infant or a mentally incompetent adult than to competent adults [48]. Most paediatric nurses with experience of JW parents who refuse blood transfusions to their children expressed worry, anger, disappointment and sadness. Over 85% of nurses believed that legal solutions that permit HCPs such procedures against parents’ will are essential and over 71% supported their implementation when necessary. Finally, while most declared that such legal procedures are essential, especially in the case of juvenile patients, more than half suggested that they be implemented only when a JW child’s life is in danger [44]. Similarly, 60% of gynaecologists and 85% of obstetricians in France reported having a protocol for managing JW women [49].

Thus, this study shows that as JW patients’ refusal of transfusions of certain blood products creates tension between such basic bioethical principles as respect for patients’ autonomy, beneficence, non-maleficence and justice [50], it often creates conflict between HCPs’ obligation to care and the duty to respect different perspectives of the patient [7, 10, 23, 51, 52]. At the same time, it shows that the ethical and legal issues arising from the case of a patient who refuses blood transfusion based on their religious beliefs are particularly conspicuous when it comes to adolescent patient. Although also in this case the legal right to refuse medical treatment based on religious beliefs is ethically justified, as it is based on respect for the individual autonomy and freedom to decide, still it rises many controversies related to the peculiarity of this period of life and the limited experience of life [53]. For that reason, because also other rights, e.g., right to drive a car, to vote or to marry, are granted at a particular age, it is often argued that because most minors do not possess fully mature decision-making capacity, adolescents should be granted the legal right to refuse life-saving or sustaining medical treatment only when they can clearly and convincingly demonstrate that they understand medical consequences of their refusal, and that their religious beliefs, which underline their decision are deeply rooted in their worldview and central to their life [54, 55].

On the other hand, even though in the HCPs’ professional opinion on the decisions made by adolescent JWs may not be in their best interests, some argue that HCPs should treat them as autonomous and competent persons able to make sound decisions for themselves and refuse treatment [56], especially since JWs are well known as “informed healthcare consumers,” and Polish legislation recognises the concept of dual consent, which involves minors older than 16 in the decision-making process related to the medical treatment. However, it also involves their legal representatives who are invited to examine the minor’s decision, and if they do not come to an agreement, the Family Court takes over and makes the consensual settlement [39, 57].

This study also shows that, while Polish NP feels unprepared when it comes to caring for JW patients, they also feel the need for improved training and legal regulation on treating patients who require non-blood management techniques. Bernaciak [58] also demonstrated that 86% of nurses receive no training whatsoever on transcultural nursing, and less than half were familiar with the concept of intercultural competence (47%). This result is in line with the observation made by Zalewska-Puchała et al. [44] that there is an urgent need for raising cultural competence in nurses working in clinical practice**.** This is particularly important in countries such as Poland, where both multidisciplinary teams and intercultural therapy are only in development, and HCPs’ awareness of non-blood management techniques and risks related to blood transfusion is inadequate. Consequently, they often lack information about so-called bloodless medicine which should be integral part of co-ordinated care, and pay insufficient attention to communication with the JW patient and their family [51, 52].

Finally, while nurses are responsible for implementing and managing blood transfusions and other blood products for patients, this research shows that taking care of JW patients who refuse such procedures raises specific medical, ethical and legal dilemmas. Although nurses are guided by the ethical imperative, i.e. the good of the patient, they are also obliged to follow the principle that requires the provision of care with respect for patients’ dignity and autonomy, regardless of their race, nationality, sex or religion. They may therefore be torn between the autonomy of patients and paternalism. This research also shows that, since NP play a fundamental role in delivering effective and safe care for JW patients, nurses should know JWs’ beliefs regarding blood transfusions and the available resources for non-blood management techniques in their healthcare institution. They should also be trained in bioethical and legal aspects of JWs’ refusal of blood transfusions.

### Limitations

Although, to the best of our knowledge, this is one of the few studies on the attitudes of Polish NP towards JWs’ right to refuse a blood transfusion, its findings are limited in several respects. Firstly, the sample was relatively small, since only two hundred and two nurses completed the questionnaire, which may have an impact on whether the results might be extrapolated and interpreted. It would therefore be desirable to compare the findings with those from a survey conducted on a larger sample size*.* Secondly, since some nurses either lacked interest in the study or were unwilling to discuss their opinions on the topic, the results represent solely the views of those nurses who agreed to participate in the study and may be extrapolated to the entire population of Polish NP only with reservations. Thirdly, due to the anonymity of our survey, it was impossible to identify those nurses who rejected the invitation. This study represents the opinions of those NP who agreed to participate in the research and more in-depth studies are required. Since this study focused on NP, future studies should compare the findings from other HCPs involved in medical care for JW patients.

Although limited by the size, scope and composition of the sample, we believe this study also enjoys some advantages that should be acknowledged. Most importantly, as there is a scarcity of previous work on the topic, this research fills the gap in research on the attitudes of Polish NP towards JWs’ refusal of blood transfusions and it may stimulate further research on the topic. By helping to understand NP’s attitudes towards JWs, this study may help identify the educational needs required to develop the intercultural competencies in nursing that should be integrated into routine hospital practices.

### Conclusion

While providing medical care NP should always act on medical knowledge and in accordance with the principles of medical ethics, and be guided by the patient’s best interests. Nurses should also acknowledge that the right of self-determination includes the right to refuse consent to recommended medical treatment even if, in the opinion of the HCPs, such a refusal may be harmful to patients or even contribute to their death. Although many nurses enrolled in this study had a moderately high level of knowledge on JWs’ stance towards blood transfusions, the majority felt unprepared to care for JW patients and lacked the cultural competencies required for caring for a patient who requires non-blood management techniques. This study therefore reveals an urgent need to train nurses in transcultural nursing and increase their cultural competencies, which should be incorporated into medical *curricula* **.**

## Data Availability

Data generated as part of this study with replication codes for all analyses are available from the corresponding author upon reasonable request.

## Abbreviations

HCPs, healthcare professionals; JWs, Jehovah’s Witnesses; NP, nursing personnel; PUMS, Poznań University of Medical Sciences.
